# The Association Between 2, 4-Dichlorophenoxyacetic Acid and Erectile Dysfunction

**DOI:** 10.3389/fpubh.2022.910251

**Published:** 2022-06-24

**Authors:** Wei Wang, Yucheng Ma, Jiawei Chen, Liao Peng, Xiaoshuai Gao, Lede Lin, Fuxun Zhang, Yang Xiong, Feng Qin, Jiuhong Yuan

**Affiliations:** ^1^Andrology Laboratory, West China Hospital, Sichuan University, Chengdu, China; ^2^Department of Urology, Institute of Urology, West China Hospital, Sichuan University, Chengdu, China

**Keywords:** erectile dysfunction, 2, 4-dichlorophenoxyacetic acid, NHANES, herbicides, men's health

## Abstract

**Background:**

2, 4-dichlorophenoxyacetic acid (2,4-D) is one of the most frequently used herbicides in the world, and it has been linked with low testosterone; however, studies regarding its effect on erectile function are limited. The current study aimed to determine the association between the 2,4-D exposure and erectile dysfunction (ED) in men from the National Health and Nutrition Examination Survey (NHANES).

**Methods:**

We analyzed data for urinary 2,4-D levels from 1,311 men (>20 years of age) in the NHANES 2001–2004. ED was assessed by a single, validated survey question. Multivariable logistic regression analysis utilizing sampling weights was performed to determine the relationship between 2,4-D exposure and ED.

**Results:**

Multivariable logistic regression models demonstrated no statistically significant association between 2,4-D exposure and ED after full adjustment [odds ratio (OR) 1.02; 95% CI 0.77–1.36; *P* = 0.882)]. Men in the 2,4-D quartile 4 groups were not associated with an increased risk of ED (OR 1.13; 95% CI 0.74–1.75; *P* for trend = 0.481). Furthermore, the association between urinary 2,4-D level and ED was not significant in the subgroup analysis stratified by age, BMI, cardiovascular disease, hypertension, diabetes, and high cholesterol.

**Conclusion:**

We demonstrated that there was no association between 2,4-D exposure and ED. Further studies are warranted to corroborate our results.

## Introduction

Erectile dysfunction (ED), defined as the persistent inability to achieve or maintain a penile erection sufficient for successful vaginal intercourse, is the most common disorder affecting middle-aged and older men (1). The prevalence rate of ED ranges from 1 to 10% in men younger than 40 years, and this number increases to 70.2% in men aged ≥70 years (1, 2). Furthermore, it is estimated that more than 322 million men globally experience ED by 2025 (3). The trends pose a great challenge for public health systems to develop policies to prevent or alleviate ED.

Herbicides are a large class of synthetic compounds that have been widely utilized to control broadleaf weeds. The use of herbicides is increasing around the world. It was estimated that the value of the worldwide herbicide market increased by 11% between 2011 and 2016 (4). 2,4-dichlorophenoxyacetic acid (2,4-D) is one of the most frequently used herbicides in the world because of its selectivity, efficiency, and low cost. In the US, the annual cost of markets for 2,4-D is nearly 57 million dollars (5). Liu et al. reported that the production of 2,4-D reached 40,000 tons in 2010 in China (6). Given the extensive application of 2,4-D, the possibility of human exposure is increasing via household and agricultural use, and consumption of food and water contaminated with 2,4-D (7). The Farm Family Exposure Study revealed that 71% of applicators had detectable 2,4-D in their urine before the application, and 100% had it in their urine (8).

Increasing evidence showed that 2,4-D exposure had adverse effects on the production and function of testosterone, which plays a critical role in male erection (9–11). Additionally, exposure to 2,4-D might increase the risks of dyslipidemia and impaired glucose metabolism that are linked to the pathogenesis of ED (12). However, literature regarding the effects of 2,4-D exposure on ED is scarce. Accordingly, in this study, we determined the association between 2,4-D exposure and ED utilizing 1,311 samples from the National Health and Nutrition Examination Survey (NHANES) 2001–2004. We hypothesized that 2,4-D exposure would be associated with the increased risks of ED.

## Methods

### Study Population

As a nationwide survey, the NHANES has been conducted every 2 years to obtain health and diet information from a representative, non-institutionalized U.S. population since 1999. This survey combines detailed in-person interviews, physical examinations, computer-based questionnaires, and laboratory tests to collect a large amount of quantitative and qualitative data. Detailed information regarding NHANES survey methods can be found at http://www.cdc.gov/nchs/nhanes/index.htm. All study protocols were approved by the ethics review board of the National Center for Health Statistics and written informed consent was obtained from all participants before any data collection.

In our analysis, we utilized NHANES data from two independent waves (2001–2002 and 2003–2004) because questionnaire information regarding ED was available only in those years. The study population was limited to those >20 years of age as the age under 20 years was not interviewed about erectile function. We excluded those participants with a history of prostate cancer because treatment for prostate cancer might be a potential iatrogenic cause of ED.

### Assessment of ED

We assessed erectile function with the following question from the Massachusetts Male Aging Study (13): “*Many men experience problems with sexual intercourse. How would you describe your ability to get and keep an erection adequate for satisfactory intercourse?*” Response options were available as “always or almost always able,” “usually able,” “sometimes able” and “never able.” ED was defined as “sometimes able” or “never able” to keep an erection adequate for satisfactory intercourse as previously validated (14). Participants who responded “almost always able” or “usually able” to maintain an erection were defined as not having ED.

### Assessment of 2,4-D Exposure

In humans, 2,4-D is primarily excreted by the kidneys with minimal metabolism, which retains its parent form in urine (15). Therefore, urine measurements can be utilized as a good indicator for 2,4-D exposure. The urinary level of 2,4-D was measured and quantified from the urine matrix of participants using an automated solid-phase extraction system. Samples were analyzed by high-performance liquid chromatography and triple quadrupole mass spectrometer with a heated electrospray ionization source (16). For 2,4-D concentrations below the limit of detection (LOD), a value of 0.15 μg/L, they were replaced with LOD divided by the square root of 2 for the purpose of increasing statistical power and precision of effects estimates (13). Additionally, we adjusted the urinary dilution by dividing each metal by the grams of creatinine per liter of urine (μg/g creatinine). All urinary metal concentrations were adjusted for log2-transformed to approximate a normal distribution.

### Covariates

Based on previous literature results, potential variables confounding the association between 2,4-D exposure and ED were included in multivariable models (17, 18). The confounders included age, race/ethnicity, body mass index (BMI), the ratio of family income to poverty, education level, marital, alcohol intake, smoking status, physical activity, cardiovascular disease, diabetes, hypertension, and high cholesterol. The ratio of family income to poverty is an index aiming to evaluate household socioeconomic status. The ratio was classified as <1.5, 1.5–3.5, and over 3.5 (19). Alcohol intake was defined as none (<1 drink per week), light (1–3 drinks per week), and heavy (≥4 drinks per week) (20). Men who smoked more than 100 cigarettes in their entire life and reported smoking every day or some days at the time of the interview were regarded as current smokers. In addition, we considered those who smoked more than 100 cigarettes in their entire life but were not smoking at the time of the interview as former smokers. Non-smokers are those who smoked <100 cigarettes during their lifetime. Physical activity status was evaluated based on responses to questions regarding whether the participants engaged in moderate or vigorous activity during the past 30 days. Participants were classified as having a history of cardiovascular disease if they reported a previous diagnosis of angina, heart attack, or coronary heart disease. Men who had a prior diagnosis of diabetes or fasting plasma glucose ≥126 mg/dl were defined as diagnosed diabetics. Four times of systolic and diastolic blood pressure on 2 separate occasions were measured from individuals. The average of those 4 measurements (≥140/90 mmHg), reporting a physician diagnosis of high blood pressure, or self-reported taking antihypertensive medication was considered hypertension. Hypercholesterolemia was defined as a total cholesterol level of 240 mg/dL or higher, a prior diagnosis of “high cholesterol,” or self-reported taking a cholesterol-lowering medication.

### Statistical Analysis

Sampling weights, strata, and primary sampling units were utilized to account for the selective bias, non-response, and over-sampling of certain subpopulations. Demographic variables were described utilizing weighted means, standard deviation, and weighted proportions. One-way ANOVA (continuous variables) and chi-square (categorical variables) tests were utilized to evaluate between-group differences. Three models were built to determine the independent association between urinary levels of 2,4-D and ED: model I, no adjustment for covariates; model II, adjusted for age, race, and BMI; model III, adjusted for age, race, the ratio of family income to poverty, education level, marital status, BMI, alcohol intake, smoking, physical activity, cardiovascular disease, diabetes, hypertension, and high cholesterol. Strengths of the association for the multivariate models were estimated by the odds ratio (OR) and their associated 95% CI.

We also conducted additional analyses. First, we categorized the urinary level of 2,4-D into four clinically relevant quartiles as ordinal categorical variables (first to fourth, setting the first as reference) to assess potential trends in the association. Second, subgroup analysis was conducted stratified by age, BMI, cardiovascular disease, hypertension, diabetes, and high cholesterol. We utilized an interaction test to assess the heterogeneity of associations between different subgroups. Third, because only 25.7 and 63% of the values were above the LOD in 2001–2002 and 2003–2004, respectively, we chose to further dichotomized urinary 2,4-D to above LOD or below LOD. Missing values were input by median (continuous) or mode (categorical) of existing cases of that variable. All statistical analyses were conducted utilizing the R-3.4.3 (http://www.R-project.org; The R Foundation, Vienna, Austria) and Empower (www.empowerstats.com, X&Y Solutions, Inc., Boston, MA). A *P*-value ≤ 0.05 was considered statistically significant.

## Results

Baseline characteristics of 1,311 participants included in this study, classified by quartile of urinary 2,4-D level, were presented in Table 1. Men in the quartile 4 group tend to be older, Mexican American or Non-Hispanic White, married or living with a partner, non-drinker, engaging in moderate physical activity, and having diabetes. In addition, compared to the *Q*1 group, men with higher urinary 2,4-D levels are more likely to have ED although did not meet the level of statistical significance (*P* = 0.064, Table 1).

**Table 1:** Baseline characteristics of the participants in the National Health and Nutrition Examination Survey (NHANES) 2001–2004.

	**Q1**	**Q2**	**Q3**	**Q4**	***P*-value**
Number	326	328	329	328	
Age, years	47.5 ± 18.8	50.4 ± 19.6	48.3 ± 18.4	53.4 ± 18.5	<0.001
Race, *n* (%)					<0.001
Mexican American	63 (19.3%)	61 (18.6%)	62 (18.8%)	78 (23.8%)	
Other hispanic	15 (4.6%)	13 (4.0%)	7 (2.1%)	9 (2.7%)	
Non-hispanic white	153 (46.9%)	183 (55.8)	186 (56.6%)	193 (58.9%)	
Non-hispanic black	86 (26.4%)	57 (17.4%)	57 (17.3%)	37 (11.2%)	
Other race	9 (2.8%)	14 (4.2%)	17 (5.2%)	11 (3.4%)	
Ratio of family income to poverty, *n* (%)					0.675
<1.5	95 (29.2%)	93 (28.5%)	101 (30.7%)	81 (24.7%)	
1.5–3.5	102 (31.3%)	106 (32.4%)	93 (28.3%)	103 (31.4%)	
Over 3.5	111 (34.0%)	106 (32.4%)	119 (36.2%)	126 (38.4%)	
Missing	18 (5.5%)	23 (7.0%)	16 (4.8%)	18 (5.5%)	
Education level, *n* (%)					0.593
Less than high school	84 (25.5%)	100 (30.5%)	96 (29.2%)	95 (29.0%)	
High school	75 (23.0%)	84 (25.6%)	74 (22.5%)	69 (21.0%)	
Above high school	168 (51.5%)	144 (43.9%)	159(48.3%)	164 (50%)	
Marital status, *n* (%)					0.006
Married or living with partner	211 (64.7%)	210 (64.0%)	237 (72.0%)	244 (74.4%)	
Living alone	115 (35.3%)	118 (36.0%)	92 (28.0%)	84 (25.6%)	
BMI, kg/m^2^, *n* (%)					0.699
BMI <20	10 (3.1%)	15 (4.6%)	16 (4.8%)	11 (3.4%)	
20 ≤ BMI <25	84 (25.8%)	86 (26.2%)	91 (27.7%)	83 (25.3%)	
25 ≤ BMI <30	136 (41.7%)	128 (39.0%)	119 (36.2%)	138 (42.1%)	
BMI ≥30	88 (27.0%)	85 (26.0%)	97 (29.5%)	88 (26.8%)	
Missing	8 (2.4%)	14 (4.2%)	6 (1.8%)	8 (2.4%)	
Alcohol intake, *n* (%)					<0.001
Non-drinker	215 (66.0%)	208 (63.4%)	247 (75.1%)	252 (76.8%)	
Light drinker	42 (12.9%)	52 (15.9%)	38 (11.5%)	21 (6.4%)	
Heavy drinker	37 (11.3%)	40 (12.2%)	26 (7.9%)	25 (7.6%)	
Missing	32 (9.8%)	28 (8.5%)	18 (5.5%)	30 (9.2%)	
Smoking, *n* (%)					0.159
Non-smoker	138 (42.3%)	130 (39.6%)	131 (39.9%)	130 (39.6%)	
Former smoker	89 (27.3%)	110 (33.5%)	112 (34.0%)	126 (38.4%)	
Current smoker	99 (30.4%)	88 (26.9%)	86 (26.1%)	72 (22.0%)	
**Physical activity status**, ***n*** **(%)**					
Moderate					0.004
Yes	155 (47.5%)	135 (41.2%)	182 (55.3%)	161 (49.1%)	
No	171 (52.5%)	193 (58.8%)	147 (44.7%)	167 (50.9%)	
Vigorous					0.656
Yes	103 (31.6%)	112 (34.1%)	116 (35.3%)	103 (31.4%)	
No	223 (68.4%)	216 (65.9%)	213 (64.7%)	225 (68.6%)	
History of cardiovascular disease, *n* (%)					0.071
Yes	22 (6.7%)	36 (11.0%)	32 (9.7%)	42 (12.8%)	
No	304 (93.3%)	292 (89%)	297 (90.3%)	286 (87.2%)	
History of diabetes, *n* (%)					0.050
Yes	35 (10.7%)	29 (8.8%)	24 (7.3%)	44 (13.4%)	
No	291 (89.3%)	299 (90.2%)	305 (92.7%)	284 (86.6%)	
History of hypertension, *n* (%)					0.904
Yes	130 (39.9%)	129 (39.3%)	139 (42.2%)	140 (42.7%)	
No	196 (60.1%)	199 (60.7%)	190 (57.8%)	188 (57.3%)	
History of high cholesterol, *n* (%)					0.350
Yes	106 (32.5%)	116 (35.4%)	117 (35.6%)	131 (40.0%)	
No	220 (67.5%)	212 (64.6%)	212 (64.4%)	197 (60.0%)	
History of erectile dysfunction, *n* (%)					0.064
Yes	85 (26.1%)	102 (31.1%)	98 (29.8%)	117 (35.7%)	
No	241 (73.9%)	226 (68.9%)	231 (70.2%)	211 (64.3%)	

*The values are presented as weighted means ± SD or unweighted counts (weighted %). BMI, body mass index; NHANSE, National Health and Nutrition Examination Survey; SD, Standard deviation*.

The association between urinary 2,4-D level and ED was positive in the unadjusted model (OR 1.27; 95% CI 1.03–1.6; *P* = 0.029, Table 2). However, this association was no longer significant after adjusting for covariates in the model I (OR 1.06; 95% CI 0.81–1.4; *P* = 0.652) and model II (OR 1.02; 95% CI 0.77–1.36; *P* = 0.882). Furthermore, we categorized the urinary level of 2,4-D into four clinically relevant quartiles as ordinal categorical variables. We found that men in the quartile 4 group were not associated with an increased risk of ED (OR 1.13; 95% CI 0.74–1.75; *P* for trend = 0.481, Table 2). As only 25.7 and 63% of the 2, 4-D values were above the LOD in 2001–2002 and 2003–2004, respectively, we chose to further dichotomize urinary 2,4-D to above LOD or below LOD. After full adjustment, no statistically significant association was observed between 2,4-D exposure and ED (OR 1.01; 95% CI 0.74–1.38; *P* = 0.943, Table 2).

**Table 2:** Association between urinary 2, 4-dichlorophenoxyacetic acid (2,4-D) and erectile dysfunction among U.S. men in the NHANES 2001–2004.

	**Crude model** **OR (95% CI), *P***	**Model 1** **OR (95% CI), *P***	**Model 2** **OR (95% CI), *P***
Continuous	1.27 (1.03, 1.60), 0.029	1.06 (0.81, 1.40), 0.652	1.02 (0.77, 1.36), 0.882
**Quartile**			
*Q*1	Reference	Reference	Reference
*Q*2	1.28 (0.91, 1.80), 0.156	1.07 (0.71, 1.64), 0.739	1.05 (0.68, 1.63), 0.830
*Q*3	1.20 (0.85, 1.69), 0.290	1.25 (0.82, 1.91), 0.296	1.20 (0.77, 1.87), 0.425
*Q*4	1.57 (1.13, 2.20), 0.008	1.17 (0.78, 1.77), 0.444	1.13 (0.74, 1.75), 0.571
*P* for trend	0.016	0.353	0.481
**Limit of detection**			
Below limit of detection	Reference	Reference	Reference
Above limit of detection	1.01 (0.80, 1.28), 0.910	1.09 (0.82, 1.46), 0.556	1.01 (0.74, 1.38), 0.943

*Crude model: non-adjusted model. Model 1: adjust for age, race, and BMI. Model 2: adjust for age, race, ratio of family income to poverty, education level, marital status, BMI, alcohol intake, smoking, physical activity, cardiovascular disease, diabetes, hypertension, and high cholesterol. BMI, Body mass index; OR, Odds ratio; CI, Confidence interval; NHANES, National Health and Nutrition Examination Survey*.

No significant difference was indicated by the interaction test between urinary 2,4-D level and ED in subgroup analysis (all *P* for interaction > 0.05, Table 3). Our results demonstrated that after full adjustment, the association between urinary 2,4-D level and ED was not significant in the subgroup analysis stratified by age, BMI, cardiovascular disease, hypertension, diabetes, and high cholesterol (Table 3).

**Table 3:** Subgroup analysis of the association between urinary 2,4-D and erectile dysfunction among U.S. men in the NHANES 2001–2004.

	**OR (95% CI)^†^**	***P*-value**	***P* for interaction**
**Stratified by age (years)**			
20–39	0.83 (0.33, 2.07)	0.692	0.130
40–59	1.14 (0.99, 1.31)	0.074	
≥60	0.85 (0.57, 1.29)	0.456	
**Stratified by BMI (kg/m** ^**2**^ **)**			
BMI <20	1.18 (0.75, 1.87)	0.467	0.682
20 ≤ BMI <25	1.30 (0.68, 2.49)	0.429	
25 ≤ BMI <30	0.87 (0.57, 1.97)	0.536	
BMI ≥30	1.06 (0.57, 1.97)	0.851	
**Stratified by cardiovascular disease**			
Yes	0.87 (0.56, 1.66)	0.678	0.179
No	1 .03 (0.75, 1.41)	0.860	
**Stratified by hypertension**			
Yes	1.20 (0.87, 1.64)	0.267	0.202
No	1.32 (0.94, 1.86)	0.110	
**Stratified by diabetes**			
Yes	1.03 (0.48, 2.22)	0.945	0.558
No	1.12 (0.84, 1.50)	0.449	
**Stratified by high cholesterol**			
Yes	1.12 (0.76, 1.67)	0.564	0.752
No	0.96 (0.66, 1.40)	0.835	

*†Adjust for age, race, ratio of family income to poverty, education level, marital status, BMI, alcohol intake, smoking, physical activity, cardiovascular disease, diabetes, hypertension, and high cholesterol. BMI, Body mass index; OR, Odds ratio; CI, Confidence interval; NHANES, National Health and Nutrition Examination Survey*.

## Discussion

This is the first epidemiologic study to determine the independent associations between 2,4-D exposure and ED. Our results demonstrated that no statistically significant association was observed between 2,4-D exposure and ED after full adjustment. Furthermore, the association between urinary 2,4-D level and ED was not significant in the subgroup analysis stratified by age, BMI, cardiovascular disease, diabetes, and high cholesterol.

In general, the application of 2,4-D in agriculture increased by 67% between 2012 and 2020, and by more than 240% between 1991 and 2020 (21). This trend continues because of unabated weed resistance. Since its first introduction on the market in the 1940 s, the 2,4-D application has dramatically increased over the next 4 decades (5). In 1996, 2,4-D consumption began to decline because of the emergence of genetically engineered glyphosate-tolerant Roundup™ soybeans and cotton (21). By the mid-2000 s, glyphosate-resistant weeds had grown and spread rapidly from farm to farm, which forced growers to add 2,4-D for weed management (21).

The increased usage of 2,4-D raises concerns regarding acute and chronic toxicity of 2,4-D exposure. Freisthler et al. revealed that the frequency of participants with high urinary 2,4-D levels increased from 17.1% in 2001–2002 to 39.6% in 2011–2012 (21). Some studies indicated that 2,4-D was associated with Parkinson's disease, autism, and non-Hodgkin lymphoma (5). Moreover, 2,4-D exposure has been linked with endocrine disruption, reproductive disorders, genetic alterations, and cancer (5). Glover et al. utilized data from 456 participants in the 2013–2014 NHANES cycle, they found a negative association between urinary 2,4-D and mean serum testosterone (β = −11.4 ng/dL, *P* = 0.02) (22). Similarly, Panuwet et al. analyzed urine and serum samples of 133 male Thai farmers and reported significant negative relationships between total testosterone and 2,4-D after controlling for covariates (11). As the principal male sex hormone, testosterone is responsible for reproduction and sexual function. Low testosterone can cause symptoms including decreased libido, fatigue, coronary artery disease, and ED (23). Nevertheless, the effects of 2,4-D exposure on ED have never been determined before. In our study, we found that the association between urinary 2,4-D level and ED was not significant after full adjustment (OR 1.02; 95% CI 0.77–1.36; *P* = 0.882). Furthermore, men in the quartile 4 group were not associated with an increased risk of ED (OR 1.13; 95% CI 0.74–1.75; *P* for trend = 0.481). These results should be interpreted with much caution as it is not generalizable for special populations exposed to higher than normal 2,4-D level. Therefore, we further performed a subgroup analysis and found that the association remained not significant in the subgroup analysis stratified by age, BMI, cardiovascular disease, diabetes, and high cholesterol.

A few limitations are present in the current study. First, the causal association between 2,4-exposure and ED cannot be examined because of the cross-sectional design of the NHANES database. Second, the diagnosis of ED was on the basis of patients' self-report, which might underestimate the actual prevalence rate of ED. However, the validity of a single-question self-report of ED has been validated previously (14). Third, a single measurement of urinary 2,4-D level might deviate from the long-term chronic exposure of 2,4-D, but Suzuki et al. suggested that metabolite levels in single spot urine could reflect longer-term exposure of subjects (24). Lastly, our study conclusions cannot be generalized because all analysis was performed only based on U. S. population, and those participants with a history of prostate cancer were excluded. Nevertheless, our study included a large-scale sample with the NHANES's rigorous quality control of the procedures. This is the first study to evaluate the relationship between 2,4-D exposure and ED. We also conducted a subgroup analysis for the sensitivity test.

## Conclusion

Utilizing a large-scale national database with rigorous quality control, our findings revealed that no statistically significant association was observed between 2,4-D exposure and ED after full adjustment. Future prospective studies are needed to validate our results.

## Data Availability Statement

Publicly available datasets were analyzed in this study. This data can be found here: NHANES.

## Ethics Statement

The studies involving human participants were reviewed and approved by National Center for Health Statistics Research Ethics Review Board. The patients/participants provided their written informed consent to participate in this study.

## Author Contributions

WW and JY proposed the conception and design. YM and JC provided administrative support. LP, XG, and LL supplied the study materials. FZ and YX collected and assessed the data. FQ analyzed the data. All authors read and approved the final manuscript.

## Funding

This work was supported by the Natural Science Foundation of China (Nos. 81871147 and 82071639) and the Sichuan Science and Technology Program (Nos. 2022YFS0028 and 2022YFS0134).

## Conflict of Interest

The authors declare that the research was conducted in the absence of any commercial or financial relationships that could be construed as a potential conflict of interest.

## Publisher's Note

All claims expressed in this article are solely those of the authors and do not necessarily represent those of their affiliated organizations, or those of the publisher, the editors and the reviewers. Any product that may be evaluated in this article, or claim that may be made by its manufacturer, is not guaranteed or endorsed by the publisher.

## Abbreviations

2, 4-D, 2: 4-dichlorophenoxyacetic acid
ED: erectile dysfunction
NHANES: national health and nutrition examination survey
LOD: limit of detection
BMI: body mass index
OR: odds ratio
CI: confidence interval.
